# Mediation role of anxiety on social support and depression among diabetic patients in elderly caring social organizations in China during COVID-19 pandemic: a cross-sectional study

**DOI:** 10.1186/s12877-023-04502-z

**Published:** 2023-12-01

**Authors:** Lanlan Zhao, Fuqin Xu, Xin Zheng, Ziwen Xu, Benjamin Osten, Kai Ji, Shuo Ding, Guoqing Liu, Shufan Yang, Ren Chen

**Affiliations:** 1 School of Health Services Management, Anhui Medical University, Hefei, 230032 China; 2 Registrars’ Department, University of Cape Coast, Cape Coast, Ghana; 3 School of Computing, Engineering and Built Environment, Edinburgh Napier University, Edinburgh, UK; 4 Research Department of Orthopaedics and Musculoskeletal Science, University College London, UCL, London, UK; 5 Key Laboratory of Public Health Social Governance, Philosophy and Social Sciences of Anhui Province, Hefei, China

**Keywords:** Anxiety, Depression, Social support, Diabetic patients, Social organizations, Elderly caring

## Abstract

**Background:**

Diabetes has become a prominent global public health problem, which is an important cause of death, disease burden, and medical and health economic burden.

Previous studies have reported that majority of persons diagnosed with diabetes later presented with psychological and mental health diseases. The study aimed to explore the mediation role of anxiety on social support and depression among diabetic patents in elderly caring social organizations (SOs).

**Methods:**

A multi-stage stratified cluster random sampling method was used in this cross-sectional study, and a questionnaire consisting of demographic questionnaire, MSPSS, GAD-7, and CES-D-10 was utilized to gather data. SPSS 22.0 and MPLUS 7.4 were used for statistical analysis. Spearman correlation analysis was employed to investigate correlations of key variables. A generalized linear model was used to exam factors associated with depression. Finally, the mediation effect among study variables was investigated by structural equation modeling (SEM).

**Results:**

The average scores of social support, anxiety, and depression were 58.41 ± 14.67, 2.95 ± 3.95, and 7.24 ± 5.53, respectively. The factors of gender, social support, and anxiety were identified as significantly influential factors related to depression among diabetic patients in elderly caring SOs. The effect of social support on depression was significantly mediated by anxiety (β = -0.467, 95%CI: -0.813 to -0.251). Furthermore, anxiety partially mediated the relationship between family support and depression (β = -0.112, 95%CI: -0.229 to -0.012), and anxiety functioned as a complete mediator in the effect of significant others' support and depression (β = -0.135, 95%CI: -0.282 to -0.024).

**Conclusions:**

The indirect effect of social support on depression through anxiety among diabetic patients in elderly caring SOs was elucidated. Social support played a key role in maintaining and regulating their mental health, particularly from family and significant others. Social support provided by both family and significant others exerted an important influence on maintaining and regulating their mental health. In light of this pathway, the elderly caring SOs should enhance the magnitude of social support from these two sources, thereby diminishing the likelihood of experiencing anxiety and depression.

## Introduction

With the progress of society and the development of medicine, the life expectancy of the population is gradually extended. Associated with this global phenomenon is the problem of aging populations. Therefore, biopsychosocial health, well-being, and quality of life of aging population are receiving increasing public and social attention. In China, in addition to providing home-based care from family, services for the elderly could be provided by nursing homes, elder care facilities, and senior apartments, which managed by governmental agencies, social organizations (SOs), or individual financiers [1, 2]. In contemporary China, due to the evolving family structure and shifting societal attitudes towards aging care, more and more older adults choose the elderly caring SOs to receive care services and support for themselves. Social organizations, also known as non-governmental or non-profit organizations, have emerged as pivotal actors within the realm of elderly care service, assuming a progressively prominent role [3].

Currently, diabetes is recognized as one of the prevalent chronic diseases globally. The incidence rate of diabetes is progressively increasing annually due to improvements in people's living standards, population aging, and the rising prevalence of obesity. According to the 10th edition of the Diabetes Atlas in 2021, the total diabetic patients around the world was 537 million, with 141 million in China [4]. In China, the prevalence of diabetes was 12.8%, indicating that approximately 1 in 10 individuals had diabetes [4]. Furthermore, diabetes is also common among residents of elderly caring SOs [5–7], and is associated with higher risk of institutionalization even after adjusting for complications [8, 9]. Health complications (stroke, blindness, renal failure, etc.) linked to diabetes include but not limited to. Those diabetic patients often experience comorbid psychological and mental diseases, with depression and anxiety emerging as the predominant conditions. Diabetes has become a prominent global public health problem, which is an important cause of death, disease burden, and economic burden. Previous studies revealed that diabetic older people in elderly caring SOs have more serious psychological problems than elderly diabetic patients who are living in personal homes [10, 11]. The group of older people with diabetes in the organizations had a lower social score in psychological domain than those who living in home through the WHOQOL questionnaire, but in physical and social domains the result was in contrast [11]. Furthermore, it is reported that the prevalence of diabetes among residents in organizations is generally on the rise [12]. More attention needs to be paid to the group of diabetic elderly who living in the elderly caring SOs.

Depression, as a severe mood disorder, can result in a notable deterioration of physical and social functioning [13]. What’s more, the psychological health of millions human around the world has been influenced by the COVID-19 pandemic, as it has intensified people's short-term and long-term stress, and damage. The World Mental Health Report released by WHO in June 2022 reported that the incidence rate of depression and anxiety increased by more than 25% in the first year of the pandemic [14]. The Chinese mental health survey in 2022, as documented in the Blue Book of Depression, unveiled a reported incidence rate of 3.4% for depression among the adult population in China. Depression stands as the primary contributor to disability among individuals diagnosed with chronic diseases. Many studies have shown that depression can affect medical symptoms related to diabetes by changing physiological processes and health-related behaviors [15, 16]. Despite the causal factor being a subject of ongoing debate, diabetic patients have an elevated risk of developing depression. Likewise, individuals with depression are more susceptible to developing diabetes [13, 17–19]. Research indicates that the incidence rate of depression in diabetic patients is twice that of the general population [20]. Besides, negative emotion associated with depression reduces the self-management ability and compliance with doctors' orders among diabetic patients. The ability to deal with interpersonal relationships and take care of themselves in life decreases, which leads to further deterioration of patients' condition [15, 21, 22]. These studies emphasize the significance of solving depression in individuals with diabetes. As a particularly vulnerable group, residents of nursing home were quarantined and socially isolated for a long time due to lockdown during the COVID-19, which lead to residents suffered from mental health problems, especially depression and anxiety [23–25].

Social support encompasses the perceived availability of social resources from individuals' social network members [2, 26]. It involves emotional, instrumental, and informational assistance provided by family, friends, and significant others, which individuals rely on to cope with stress, solve problems, and meet various needs. Social support has gained increasing recognition as a crucial determinant in promoting the well-being of diabetic patients, which can effectively reduce patients’ depression in nursing home [27, 28]. Earlier researches suggest that lower social support was related to depression, and poor adherence and glycemic control of diabetes [28–30], all of which contributed to worsening the biopsychological health of diabetic patients. Besides, social support from family was important to improve patients’ adherence [31].

In addition to depression, anxiety is another common complication of individuals with diabetes [32, 33]. The increased anxiety among diabetic patients can occur at the moment of being diagnosed, onset of complications, and the fear of hypoglycemia [32]. Overall, our study suggest that diabetes is associated with an increased risk of anxiety. A prior investigation revealed that up to 90% of individuals with anxiety disorders experience comorbid depression [34]. This has been corroborated another investigation which demonstrated that 39% of individuals diagnosed with anxiety met the criteria for depression [35]. Many studies have consistently shown an inverse relationship between anxiety and social support [2, 36]. Nevertheless, the relationship between social support and anxiety among residents with diabetes in elderly caring SOs remains unclear.

A number of researches has explored the relationship among social support, anxiety, and depression [37–39]. While most studies have investigated how social support mediates the association between anxiety and depression [40, 41], limited research has focused on understanding how anxiety influences the link between social support and depression specifically in diabetic patients. Nonetheless, some studies have provided evidence of a positive correlation between depression and anxiety in individuals diagnosed with type 2 diabetes, while depression is negatively correlated with social support [38]. Given recent research findings suggesting that anxiety played a predictive role in depression and the protective mechanism of social support in anxiety and depression [2, 42–44], we aim to examine the mediation role of anxiety on social support and depression among diabetic patents in elderly caring SOs.

**Fig. 1:**
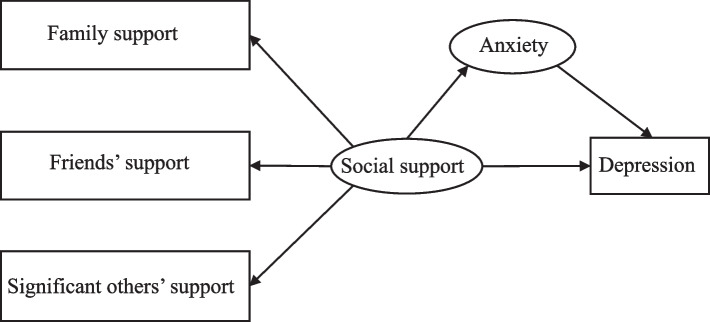
Theoretical model

Therefore, we proposed the following hypotheses (Table 1) in alignment with the theoretical model (Fig. 1). To prove the above research hypotheses, structural equation model (SEM) was used. Finally, we aim to provide guidance for implementing intervention measures aimed at enhancing the biopsychosocial health of residents with diabetes in organizations.

**Table 1:** Seven hypotheses among diabetic patients

Hypotheses
**H1:** Social support was negatively correlated with depression
**H2:** Anxiety was positively correlated with depression
**H3:** Social support was negatively correlated with anxiety
**H4:** Anxiety played a mediating role between social support and depression
**H4-a:** Anxiety played a mediating role between family support and depression
**H4-b:** Anxiety played a mediating role between friends’ support and depression
**H4-c:** Anxiety played a mediating role between significant others’ support and depression

## Methods

### Data and population

This study was carried out in July and August 2022 in Chongqing Province, China. A multi-stage stratified cluster random sampling method was employed to select participants [2], and data collection was conducted through a structured questionnaire. A total of 80 elderly caring SOs were selected from 4 districts (D, J, T, and R) in Chongqing province. Through the health records of the elderly caring SOs, which contained information on residents’ chronic diseases, potential participant diagnosed with diabetes in secondary hospitals and above were identified. Following that, face-to-face interviews were conducted. The objective and procedure of this survey were explained to the interviewees, and then informed consent was obtained before starting the survey. The inclusion criteria included (1) age 60 years or over, (2) diagnosed with diabetes in secondary hospitals and above (3) willingness to participate in this study. The exclusion criteria included (1) could not effectively communicate with investigators resulting from organic mental conditions such as visual impairments, auditory impairments, and so on (2) unwillingness to participate in this study. What’s more, our survey team was composed of members from Chongqing Medical University and Anhui Medical University who were well-trained and skilled. In total, 205 completed and valid questionnaires were analyzed.

## Measures

### Demographic characteristics

Demographic data includes gender, age, marital status, education, whether visited by relatives, and self-reported institutional satisfaction. Age was divided into ≤ 74, 75–89, and ≥ 90 years. Marital status was categorized into three groups: married (with spouses), single, and other (including widowed, divorced, or other). Education was classified into four groups: uneducated, primary school, middle school, and high school and above. Whether visited by relatives was divided into yes, no. Self-reported institutional satisfaction was sorted into low, medium, and high.

### Multidimensional Scale of Perceived Social Support (MSPSS)

Social support was assessed based on three informal sources (family, friends, and significant others) using MSPSS [45]. The MSPSS has demonstrated strong internal consistency and good reliability in previous research [2, 46]. The instrument was composed of 12 self-assessment items, with each item rated on a seven-point Likert scale. Consequently, the total score of MSPSS ranged from 12 to 84, with a higher score indicating greater social support. In this survey, the Cronbach's α for the total scale and its subscales were as follows: 0.923 for social support, 0.956 for family support, 0.952 for friends’ support, and 0.921 for significant others’ support.

### The 7-item self-reported Generalized Anxiety Disorder Scale (GAD-7)

Anxiety was gathered with GAD-7 [47, 48]. Respondents were asked to answer 7 items on a 4-point scale by considering the last 2 weeks. Multiple studies have demonstrated the acceptable internal consistency and good reliability of the GAD-7 [45, 49]. Cronbach’s α of this scale among current sample was 0.906. The GAD-7 yielded a total score ranging from 0 to 21, with higher scores indicating greater anxiety. Additionally, the cut-off scores of 5, 10, and 15 are commonly used to identify mild, moderate, and severe levels of anxiety symptoms, respectively [49].

### The 10-item Center for Epidemiologic Studies Depression Scale (CES-D-10)

Depression was measured by CES-D-10 [50], which was a validated instrument used in China [51]. Extensive research has provided evidence of this scale's good reliability and validity [52]. Participants were asked to answer 10 items on a 4-point scale. The response options for the 8 negative items were scored on a scale ranging from 0 = “rarely or none of the time” (< 1 day) to 3 = “most or all of the time” (5–7 days), while the scoring of the response options for the 2 positive items was reversed. A week recall period was used. Thus, the total score of this scale fell within the range of 0 to 30, with higher scores indicating greater depression. The reliability of CES-D-10 in this study was good with Cronbach's α yielded a coefficient of 0.814.

### Statistical analysis

SPSS 22.0 and MPLUS 7.4 were used for statistical analysis. First, descriptive statistics were employed to provide an overview of participant characteristics. Second, Spearman correlation analysis was conducted among study variable. Third, a generalized linear model was utilized to find the important influencing factors of depression and exam the relationship among social support, depression, and anxiety. Lastly, SEM was conducted to examine our seven hypotheses, while controlling significant influencing variables. The total score of social support and anxiety were treated as latent variables, while depression was measured as a measurement variable. In this study, *p* < 0.05 was considered significant.

In the structural equation modeling, bootstrap analysis was used. 95% bias-corrected confidence intervals for the indirect effect and moderated mediation effect from 1000 resamples of the data were produced by bootstrap analysis [53]. The model fit was considered acceptable if χ^2^ / df ≤ 3, CFI ≥ 0.90, TLI ≥ 0.90, SRMR ≤ 0.08, and RMSEA ≤ 0.10 [54, 55].

## Results

### Descriptive analysis

The average age of the 205 diabetic patients residing in elderly caring SOs was 78.37 ± 9.39 years, 53.20% were female, and 61.50% were widowed, divorced or other. Majority of the respondents were visited by their relatives (75.10%), and 35.6% of them had primary school level education. Furthermore, the mean score of anxiety, social support, and depression were 2.95 ± 3.95, 58.41 ± 14.67, and 7.24 ± 5.53, respectively (Table 2).

**Table 2:** Description of the sample (*n* = 205)

Variables	n (%), Mean ± SD
**Gender**	
Male	96 (46.80)
Female	109 (53.20)
**Age (years)**	
≤ 74	61 (29.80)
75–89	127(62.00)
≥ 90	17 (8.30)
**Marital status**	
Married have spouses	39 (19.00)
Single	40 (19.50)
Widowed, divorced or other	126 (61.50)
**Education**	
Uneducated	65 (31.70)
Primary school	73 (35.60)
Middle school	34 (16.60)
High school and above	33 (16.10)
**Whether visited by relatives**	
No	51 (24.90)
Yes	154 (75.10)
**Self-reported institutional satisfaction**	
Low	51 (24.90)
Medium	98 (47.80)
High	56 (27.30)
**Anxiety**	2.95 ± 3.95
**Social support**	58.41 ± 14.67
Family support	20.22 ± 6.95
Friends’ support	18.45 ± 6.42
Significant others’ support	19.74 ± 4.95
**Depression**	7.24 ± 5.53

### Correlation among study variables

Spearman correlation analysis revealed a significantly negative association between anxiety and social support, along with its three dimensions (Table 3). Nevertheless, a positive correlation was found between anxiety and depression. Likewise, the three dimensions of social support showed positive correlations with each other.

**Table 3:** The correlation among study variables

Variables	1	2	3	4	5	6
1. Anxiety	1					
2. Social support (total)	-0.265^**^	1				
3. From family	-0.172^*^	0.748^**^	1			
4. From friends	-0.249^**^	0.834^**^	0.358^**^	1		
5. From significant others	-0.282^**^	0.823^**^	0.462^**^	0.761^**^	1	
6. Depression	0.683^**^	-0.355^**^	-0.280^**^	-0.305^**^	-0.267^**^	1

^*^p < 0.05; ^**^p < 0.001

### A generalized linear model

Model 1 analyzed the effects of anxiety and social support on depression. Table 4 revealed that gender was a significantly influential factor of depression among diabetic patients in elderly caring SOs, in addition to social support and anxiety. Furthermore, females (β = 1.273, *p* = 0.044) displayed elevated levels of depression compared to males. Model 2 analyzed the effect of anxiety and the three subscales of social support on depression. The findings indicated a significant association between depression and factors such as gender, family support, friends' support, and anxiety. Additionally, family support (β = -0.130, *p* = 0.018) exhibited a negative correlation with depression, as well as friends' support (β = -0.118, *p* = 0.045), while anxiety (β = -0.867, *p* < 0.001) was positively associated with depression. However, support from significant others had no significant correlation with depression.

**Table 4:** Results from the generalized linear model analysis of depression (*N* = 205)

Variables	Model 1			Model 2		
	**B (SE)**	**95%CI**	***p***	**B (SE)**	**95%CI**	***p***
Intercept	9.015 (1.624)	5.833, 12.197	< 0.001**	8.780 (1.665)	5.516, 12.043	< 0.001**
**Social support (total)**	-0.079 (0.022)	-0.121, -0.036	< 0.001**			
Family support				-0.133 (0.056)	-0.242, -0.024	0.017*
Friends’ support				-0.118 (0.060)	-0.236, -0.000	0.049*
Significant others’ support				0.020 (0.084)	-0.145, 0.185	0.816
**Anxiety**	0.881 (0.074)	0.735, 1.026	< 0.001**	0.882 (0.074)	0.737, 1.027	< 0.001**
**Gender**						
Male (ref)						
Female	1.308 (0.641)	0.053, 2.564	0.041*	1.321 (0.644)	0.060, 2.582	0.040*
**Age (years)**						
≤ 74 (ref)						
75–89	0.292 (0.657)	-0.997, 1.580	0.657	0.209 (0.663)	-1.091, 1.509	0.752
≥ 90	0.776 (1.106)	-1.391, 2.943	0.483	0.698 (1.103)	-1.464, 2.860	0.527
**Marital status**						
Married have spouses (ref)						
Single	-1.144 (1.038)	-3.178, 0.890	0.270	-1.297 (1.059)	-3.371, 0.778	0.221
Widowed, divorced or other	-0.541 (0.722)	-1.957, 0.874	0.453	-0.446 (0.723)	-1.862, 0.971	0.537
**Education**						
Uneducated (ref)						
Primary school	0.704 (0.670)	-0.608, 2.017	0.293	0.679 (0.669)	-0.632, 1.990	0.310
Middle school	0.645 (0.831)	-0.983, 2.273	0.438	0.627 (0.828)	-0.995, 2.249	0.449
High school and above	0.217 (0.866)	-1.480, 1.914	0.802	0.304 (0.866)	-1.393, 2.002	0.725
**Whether visited by relatives**						
No (ref)						
Yes	-0.981 (0.800)	-2.549,0.588	0.220	-0.715 (0.824)	-2.330, 0.901	0.386
**Self-reported institutional satisfaction**						
Low (ref)						
Medium	0.140 (0.670)	-1.173, 1.452	0.835	0.085 (0.668)	-1.225, 1.395	0.899
High	0.398 (0.772)	-1.116,1.912	0.606	0.270 (0.775)	-1.249, 1.789	0.728

*p < 0.05; **p < 0.001

### Structural equation modeling

**Fig. 2:**
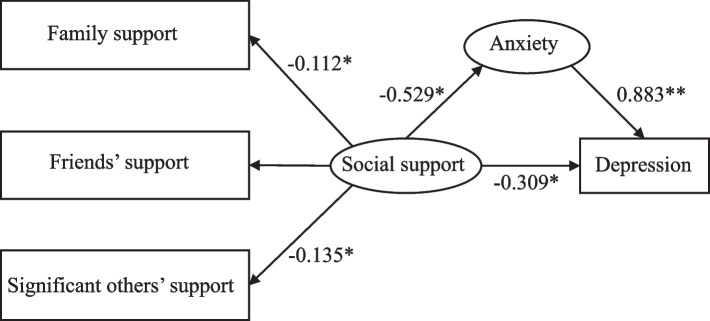
Structural equation model among social support, anxiety, and depression *p < 0.05; **p < 0.001

Under the condition of controlling variables that have a significant influence on the study variables, models were built to examine the mediation role of anxiety on social support and depression among diabetic patients in elderly caring SOs (Fig. 2). The model demonstrated good fit: χ2/df = 2.70, CFI = 0.929, TLI = 0.910, SRMR = 0.057, RMSEA = 0.091.

Figure 2 and Table 5 present the standardized direct and indirect effects among three key variables. Depression exhibited a significantly negative correlation with social support (β = -0.309, 95%CI: -0.618 to -0.010), thus, supporting Hypothesis 1. The findings of the study indicated that anxiety had a significantly positive impact on predicting depression (β = 0.883, 95%CI: 0.679 to 1.149), corroborating Hypothesis 2. Social support exhibited a significantly negative predictive effect on anxiety (β = -0.529, 95%CI: -0.921 to -0.275), which support Hypothesis 3. After including the mediator variable (anxiety), social support showed a significant adverse effect on depression (β = -0.467, 95%CI: -0.813 to -0.251), thereby supporting Hypothesis 4. Moreover, anxiety accounted for 60.18% of the mediated effect (-0.467/-0.776).

**Table 5:** Bootstrap analysis of mediation effect test

Path	Effect	Estimate	SE	95%CI	Mediating effect (%)
Social support → Depression	Total effect	-0.776**	0.182	-1.206, -0.459	60.18%
	Direct effect	-0.309*	0.153	-0.618, -0.010	
	Indirect effect	-0.467*	0.140	-0.813, -0.251	
Family support → Depression	Total effect	-0.252*	0.082	-0.412, -0.085	44.44%
	Direct effect	-0.140*	0.068	-0.273, -0.011	
	Indirect effect	-0.112*	0.055	-0.229, -0.012	
Significant others’ support → Depression	Indirect effect	-0.135*	0.065	-0.282, -0.024	100%

*p < 0.05; **p < 0.001

In the further analysis, mediation of anxiety was performed between the three subscales of social support and depression. The model showed good fit: χ2/df = 2.025, CFI = 0.955, TLI = 0.940, SRMR = 0.034, RMSEA = 0.071. As shown in Table 5, anxiety partially mediated the relationship between family support and depression (β = -0.112, 95%CI: -0.229 to -0.012), and anxiety accounted for 44.44% of the mediated effect of family support on depression (-0.112/-0.252). Therefore, Hypothesis 4-a was confirmed. In addition, anxiety played a full mediating role in the effect of significant others’ support and depression (β = -0.135, 95%CI: -0.282 to -0.024), which confirmed Hypothesis 4-c.

## Discussion

Diabetes has become a prominent global public health problem, which is related to biopsychological diseases such as depression and anxiety [32]. Especially amid the COVID-19 pandemic, the residents in elderly caring SOs, as a particularly vulnerable group, are at a strong risk of experiencing psychological diseases due to diabetes and social isolation [23, 24]. The study was dedicated to explore the mediating role of anxiety in the relationship between social support (including its three subscales) and depression among diabetic patents in elderly caring SOs. The results revealed that compared to diabetic patients in the community, the diabetic patents in elderly caring SOs targeted in this study had lower overall social support, with consistent results in the family support and friends’ support dimensions. However, in the dimension of support from significant others, diabetic patents in elderly caring SOs had higher levels of support, which may be due to their friendly interactions with staff and other companions in the organizations, receiving emotional and practical assistance [56]. The level of social support in the population under investigation in this study was moderately satisfactory [45, 57]. A study from Sichuan, China, also indicated a moderate degree of social support among diabetic patients [27]. Their anxiety levels in this study were relatively mild, which was lower than the anxiety levels observed among diabetes patients in a hospital in Nepal [58]. In terms of depressive symptoms, they displayed either no symptoms of depression or extremely mild depressive symptoms, which was lower than the estimates reported in another study [59]. The differences in depressive and anxiety conditions may be attributed to the varying environments in which diabetes patients are situated. More importantly, during the COVID-19, family members and significant others might lose the opportunity for in-person visits, while the frequency of communication through alternative means such as phone calls and video chats may be higher. This may reduce their level of anxiety and depression. Additionally, compared to usual circumstances, the employees of elderly caring SOs would place greater emphasis on the health conditions of the residents [60, 61].

Based on the study findings, gender emerges as a predictive factor for depression among the population targeted in this study, with female patients being more susceptible to experiencing depressive symptoms. This phenomenon has been consistently documented in previous studies, suggesting a greater incidence of depression among females in elderly caring SOs compared to their male counterparts [62–65]. One possible explanation is that female diabetic patients residing in elderly caring SOs may have limited social interactions, leading to a stronger desire to share their emotions. Due to their usual residence in such facilities and relatively less attention from family members, combined with restricted visitation during the pandemic, the female diabetic patients perhaps were more inclined to seek "help". Thus, amplifying the pain caused by their illness in the hope of receiving understanding and assistance from others [66].

Under the controlled conditions of influential variables, we have identified the following connections among the core variables. Firstly, a negative association was found between social support and depression, suggesting that targeted people in this study who exhibit lower levels of social support are more prone to experiencing heightened levels of depression. This finding aligns with preceding research findings [67–69]. This research also found a positive correlation between anxiety and depression, highlighting the crucial function of anxiety in this context [70]. Interestingly, we observed that an even more remarkable finding is that we have also discovered that social support did not only directly influences depression but also exerted an indirect influence through anxiety, thereby establishing an indirect negative correlation mediated by anxiety. This aligns with the results presented by Ji Kai in his study [44, 49]. Our study has unveiled a novel pathway through which social support alleviates depression by means of anxiety. This discovery opens up new avenues for shaping the trajectory of late-life health and well-being. From a practical standpoint, elderly caring SOs should place greater emphasis on the extent to which social support meets the companionship and support needs of diabetic patients. It is crucial to promptly identify anxiety emotions within the population and actively guide individuals to prevent the escalation of depressive conditions on a larger scale [71, 72].

Importantly, the further mediation analyses conducted on the three dimensions of social support showed that lower levels of support from family members and significant others act as catalysts for depression. This effect is mediated through anxiety, resulting in an elevation of depressive symptoms. Moreover, it was found that support from significant others exerted influence on depression exclusively through anxiety. This finding is consistent with the research conducted by Rehan Aziz, who identified social support, diabetes, and anxiety as factors influencing depression [63]. Relatedly, in a study focusing on perinatal depression among women, it was found that inadequate support from partners, friends, and family members was linked to a higher likelihood of experiencing depression and anxiety [73]. This might be because family members and significant others can help individuals build a strong support network, which contributes to the prevention of depression. They can provide emotional support, encouragement, and understanding, making individuals feel cared for and supported. This helps reduce anxiety about illness and life, thus reducing the occurrence of depressive symptoms. Additionally, family members and significant others can provide information and education about mental health and depression. Understanding the symptoms and coping methods of depression can help individuals better understand their own situation and take early action.

In order to effectively reduce depression levels among the targeted people in this study and enhance their overall quality of life in later stages, we propose the following recommendations. Firstly, leveraging advancements in modern technology, such as remote video communication, home network connectivity devices, and digital photo albums, can bridge the gap between diabetic patients and their family members. Secondly, we suggest incorporating an innovative theory, namely the Act-Belong-Commit mental health promotion campaigns. Act-Belong-Commit is a mental health promotion campaign aimed at enhancing the mental health of individuals and communities. By encouraging people to actively engage in sports, artistic activities, volunteer work, social interactions, and other activities beneficial to mental health, Act-Belong-Commit aims to foster meaningful relationships and community involvement to help individuals and communities establish positive mental health habits and lifestyles. These activities are crucial for enhancing social support and reducing feelings of loneliness, ultimately facilitating social connections among diabetic patients with emotional issues [71]. This applies to elderly caring social SOs, where it is recommended that nursing homes facilitate a wide range of cultural and recreational activities. Collaboration with other institutions can be considered to foster the integration of social networks among individuals with diabetes. Thirdly, the elderly caring SOs should collaborate with the government, medical institutions, and other relevant organizations to provide comprehensive services that integrate elderly care, medical services, and nursing, aiming to achieve a transition towards an integrated model of medical and elderly care. This will help establish a new type of elderly caring SOs. Fourthly, to further alleviate the anxiety of diabetic patients in elderly caring SOs, we encourage elderly who can exercise independently to engage in moderate physical activity every day, including walking, yoga, Tai Chi, and light running. For elderly with physical limitations, we suggest fully utilizing the volunteer resources of the organization to channel their passive emotions. Finally, we hope that the government can further fulfill its leading role by collaborating with social organizations and private capital to promote the high-quality development of elderly care institutions. This includes meeting the diverse care needs of the service recipients and gradually addressing their psychological and emotional needs.

We acknowledge this study has some limitations. Firstly, our research design employed a cross-sectional approach, focusing only on diabetic patients in specific elderly caring SOs in Chongqing during COVID-19 pandemic period. As a result, the causal inference capability of the study findings is limited. Secondly, our study was conducted solely in Chongqing, which poses challenges in generalizing the research results to other populations. In future research, we aim to address these limitations and further enhance the study's scope.

## Conclusion

Our study suggests that gender, social support, and anxiety can all be factors influencing depression among diabetic patients in elderly caring SOs. Simultaneously, our study is a significant step towards clarifying the important role of anxiety in mediating the relationship between social support and depression, especially social support from family and significant others, which offers fresh perspectives on improving targeted people’s quality of life. The elderly caring SOs should actively engage in collaborative activities, expand the social network of diabetic patients, enhance the magnitude of social support, and further alleviate anxiety levels, thereby diminishing the likelihood of experiencing depression.

## Data Availability

The datasets generated and analyzed during the current study are available from the corresponding author on reasonable request.

## Abbreviations

S.E.: Standard error
CI: Confidence interval
Ref: Reference
